# The Immediate Effect of COVID-19 Vaccination on Anticoagulation Control in Patients Using Vitamin K Antagonists

**DOI:** 10.1055/s-0042-1742628

**Published:** 2022-03-04

**Authors:** Chantal Visser, Joseph S. Biedermann, Melchior C. Nierman, Felix J.M. van der Meer, Anouk J.W. Gulpen, Yvonne C.F. Moors, Suzanne C. Cannegieter, Willem M. Lijfering, Marieke J.H.A. Kruip, on behalf of the Dutch COVID & Thrombosis Coalition

**Affiliations:** 1 Department of Hematology, Erasmus MC, Erasmus University Medical Center Rotterdam, Rotterdam, The Netherlands; 2 Department of Thrombosis and Anticoagulation, Atalmedial Medical Diagnostics Centers, Amsterdam, The Netherlands; 3 Department of Internal Medicine, Section of Thrombosis and Hemostasis, Leiden University Medical Centre, Leiden, The Netherlands; 4 Department of Internal Medicine, Elkerliek Hospital, Helmond, The Netherlands; 5 Department of Clinical Epidemiology, Leiden University Medical Center, Leiden, The Netherlands; 6 Kennisinstituut van de Federatie Medisch Specialisten, Utrecht, The Netherlands; 7 Thrombosis Service Star-shl, Rotterdam, The Netherlands

**Keywords:** acenocoumarol, phenprocoumon, BNT162b2 vaccine, COVID-19 vaccines, anticoagulants

## Abstract

**Background** In January 2021, the Dutch vaccination program against severe acute respiratory syndrome coronavirus 2 (SARS-CoV-2) was started. Clinical studies have shown that systemic reactions occur in up to 50% of vaccine recipients. Therefore, COVID-19 vaccination could affect anticoagulation control, potentially leading to an increased risk of thrombotic events and bleeding complications.

**Aims** This article investigates whether the BNT162b2 vaccine affects anticoagulation control in outpatients using vitamin K antagonists (VKAs).

**Methods** A case-crossover study was performed in a cohort of outpatient VKA users from four Dutch anticoagulation clinics who received a BNT162b2 vaccine. International normalized ratio (INR) results and VKA dosages before the first vaccination, the reference period, were compared with those after the first and second vaccination.

**Results** A total of 3,148 outpatient VKA users were included, with a mean age (standard deviation) of 86.7 (8.7) years, of whom 43.8% were male, 67.0% used acenocoumarol, and 33.0% phenprocoumon. We observed a decrease of 8.9% of INRs within range in the standard intensity group (target INR 2.0–3.0). There was both an increased risk of supratherapeutic (odds ratio [OR] = 1.34 [95% confidence interval [CI] 1.08–1.67]) and subtherapeutic levels (OR = 1.40 [95% CI 1.08–1.83]) after first vaccination. In the high-intensity group (target INR 2.5–3.5), the risk of a supratherapeutic INR was 2.3 times higher after first vaccination (OR = 2.29 [95% CI 1.22–4.28]) and 3.3 times higher after second vaccination (OR = 3.25 [95% CI 1.06–9.97]).

**Conclusion** BNT162b2 was associated with an immediate negative effect on anticoagulation control in patients treated with VKAs, so it is advisable to monitor the INR shortly after vaccination, even in stable patients.

## Graphical abstract

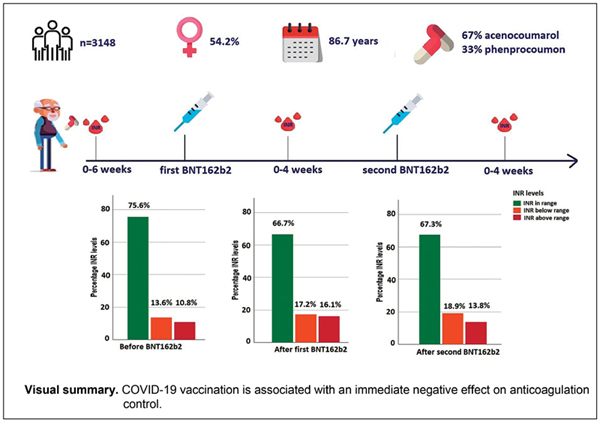

## Introduction

The novel coronavirus infection disease (COVID-19), first identified in December 2019 in Wuhan, 1 China, has contributed to significant morbidity and mortality worldwide, with the number of new cases still increasing. 2 As of the first of July 2021, almost 200 million individuals worldwide have tested positive for severe acute respiratory syndrome coronavirus 2 (SARS-CoV-2). 2 The virus can lead to various disease states, from a mild flu-like illness to very severe pneumonia with profound hypoxemia requiring mechanical ventilation. 3 4 In addition to primarily affecting the respiratory system, several studies have reported effects of SARS-CoV-2 on coagulation and the cardiovascular system. 5 6 7 8 COVID-19 infection has been associated with elevated D-dimers, high fibrinogen levels, and slightly prolonged prothrombin time. 5 This coagulopathy is one of the most distinct prognostic factors of poor outcome in patients with COVID-19 7 8 and it is associated with both arterial and venous thrombotic events. 9

COVID-19 coagulopathy could potentially affect the therapeutic stability in patients treated with vitamin K antagonists (VKAs). Treatment with VKA poses various difficulties because of their pharmacological properties. These properties include a slow onset of action and numerous interactions with dietary intake and medication. 10 For instance, medications affecting albumin binding or cytochrome 450 isoenzymes, as well as dietary vitamin K, can offset the effect of VKAs. 11 Regular measurements of the international normalized ratio (INR) are required to monitor the anticoagulant effect. Another problem is the narrow therapeutic range of VKA. Any deviation can have potentially deleterious effects, such as thrombotic events or bleeding complications. These events can be prevented by correctly dosing the INR inside the therapeutic range. 12

Any systemic event such as illness, fever, or physical stress can influence a patient's INR, hence contributing to a higher risk of major events, 13 as was recently shown in VKA users infected with SARS-CoV-2. 14 So COVID-19 could potentially lead, also indirectly, to an increased risk of thrombotic events, bleeding, or death in patients treated with VKAs. In January 2021, the Dutch vaccination program against SARS-CoV-2 was started, mainly using BNT162b2 15 (Pfizer/BioNTech) vaccine in the elderly and people with underlying medical conditions, including many VKA users.

COVID-19 vaccination can also potentially affect anticoagulation control by directly or indirectly influencing the INR and thereby decreasing the therapeutic stability due to the abovementioned effects of SARS-CoV-2 on coagulation. Besides, clinical studies have shown that systemic reactions, including fever and chills, occur in up to 50% of vaccine recipients, depending on the type of vaccine used. 15 16 17 It is known that systemic reactions such as fever can alter the therapeutic stability in VKA users. 13 18 Therefore, we aim to investigate whether the BNT162b2 vaccine affects anticoagulation control in patients using VKAs. To this end, we have performed a case-crossover study in a cohort of VKA users from four anticoagulation clinics in the Netherlands.

## Methods

### Study Design

**Fig. 1:**
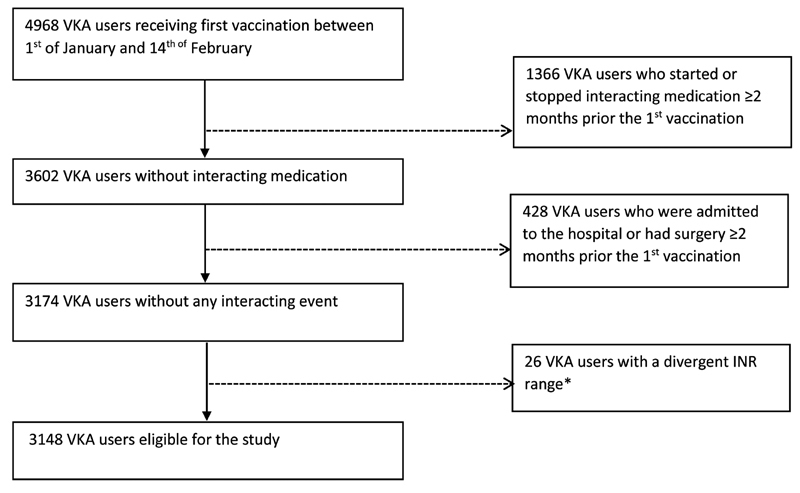
Flow diagram of eligible vitamin K antagonist (VKA) users. *A divergent international normalized ratio (INR) range is defined as any therapeutic target range, which differs from 2.0 to 3.0 and 2.5 to 3.5.

In this case-crossover study, we included all adult outpatient VKA users treated by four Dutch anticoagulation clinics, namely Atalmedial, trombosedienst Leiden, Star-shl, and Elkerliek trombosedienst, who received a BNT162b2 vaccine. We included outpatient VKA users who received at least one vaccine between January 1 and February 14, 2021. VKA users were excluded when 3 months before until the end of the study they (1) had been hospitalized or had received a surgical intervention, (2) started or stopped any medication interacting with VKA, (3) had a deviant INR range (e.g., 3.0–4.0 or 1.5–2.0), or (4) switched from acenocoumarol to phenprocoumon, or vice versa ( Fig. 1 ). The national list of medication interacting with VKA established by the Federation of Dutch anticoagulation clinics 19 was used to identify any interacting medication.

The Erasmus University Medical Centre's ethics committee granted a waiver for informed consent because of the study's retrospective nature.

### Data Collection

We retrieved data from electronic patient files including baseline characteristics, year of VKA initiation, indication for VKA treatment, INR target range and INR results, and VKA dosages. Other collected data were surgical interventions, hospital admissions, registered complications, and medication.

At the abovementioned anticoagulation clinics, all VKA users are strictly monitored at least once every 6 weeks. During each patient visit, changes in comedication, bleeding events, scheduled surgical interventions, hospital admissions, and onset of comorbidities are documented, along with the date and type of the received vaccination. The anticoagulation clinics were encouraged to measure the INR within 2 weeks after vaccination.

### Outcome Measures

Our main outcome was the percentage (%) of sub- and supratherapeutic INR after both vaccinations. We used the most recent INR measured prior to vaccination and the first INRs measured after both vaccinations. The VKA users were divided into a standard (therapeutic INR range 2.0–3.0) or high-intensity (therapeutic INR range 2.5–3.5) treatment group. For both groups, the percentage of INR results below, within, or above therapeutic range were determined prior to vaccination and after both vaccinations. We used the percentage (%) of INRs ≥ 5 as a surrogate marker for bleeding complications because of the heterogeneity between anticoagulation clinics in registering complications. An INR ≥ 5 is associated with a higher risk of bleeding complications 20 and will function as a surrogate marker for bleeding complications. The percentage of INRs ≥ 5 prior to vaccination and after the first and the second vaccination was compared. Finally, we studied the effect of both the first and second vaccination on the mean INR and VKA dosage and the percentage of INR results followed by a significant dose adjustment. A significant dose adjustment was defined as any dose adjustment of 10% or more.

### Statistical Analysis

Data for continuous variables were expressed as means with standard deviation (SD) or median with interquartile range depending on the normality of the distribution. We expressed categorical data as numbers with percentages. The reference categories in all analyses were the INR and VKA dosage at the last known date before vaccination. In this study, VKA users were compared with themselves (crossover analysis). We compared absolute differences in INR and VKA dosage using paired *t* -test or a Wilcoxon signed-rank test in case of a normal distribution or skewed distribution, respectively. Percentages were compared using McNemar's test. Conditional logistic regression was used to calculate odds ratios (ORs). Subgroup analyses were performed restricting to patients with an INR within range, patients with a measurement ≤ 14 days after vaccination, and VKA users with a poor “time in therapeutic range” (TTR), defined as a TTR < 60%. The TTR was calculated using the Rosendaal method. 21 To verify our results, we performed several sensitivity analyses. First, we replaced the most recent INR before vaccination with the second INR before vaccination as baseline. Second, we replaced the most recent INR with the INR measured 1 to 2 months before vaccination as baseline. If this INR was the second INR before vaccination, we included the INR before the second INR. The most recent INR before vaccination as baseline could be influenced as professionals might wait for the optimal INR to vaccination. We also selected the 20% most stable patients based on the TTR of the previous 6 months. We excluded the most recent INR before vaccination as this could potentially influence the TTR. Furthermore, we stratified by type of VKA (acenocoumarol or phenprocoumon), because phenprocoumon is associated with better anticoagulation control. 22 Finally, we stratified by therapeutic range, as anticoagulation control is higher in the standard intensity group than in the high-intensity group. 23 All statistical analyses were performed with IBM SPSS statistics version 25.

## Results

In total, 4,995 outpatient VKA users received their first BNT162b2 vaccine during the study period. After the exclusion criteria were applied, 3,148 outpatients were included ( Fig. 1 ).

Of these 3,148 patients, the mean age (SD) was 86.7 (8.7) years. Note that 43.8% were male, 67.0% used acenocoumarol, 33.0% used phenprocoumon, and 8.8% had an INR target range between 2.5 and 3.5. Table 1 shows the patient characteristics at baseline by VKA type. Phenprocoumon users were significantly younger than the acenocoumarol users (mean age [SD] 85.9 [9.1] vs. 87.1 [8.4], *p* < 0.001). All other clinical characteristics were similar between both groups.

**Table 1:** Clinical characteristics of all vaccine recipients

	Phenprocoumon	Acenocoumarol
Patients ( *n* , %)	1,040 (33.0)	2,108 (67.0)
Age (SD)	85.93 (9.1) b	87.14 (8.4) b
Male ( *n* , %)	465 (44.7)	915 (43.4)
Treatment indication a		
Atrial fibrillation ( *n* , %)	828 (79.6)	1,700 (80.6)
Venous thrombosis ( *n* , %)	98 (9.4)	177 (8.4)
Mechanical heart valves ( *n* , %)	37 (3.6)	100 (4.7)
Vascular surgery ( *n* , %)	19 (1.9)	35 (1.7)
Ischemic heart disease ( *n* , %)	5 (0.5)	13 (0.6)
Other ( *n* , %)	53 (5.1)	83 (3.9)
Target INR		
[2.0–3.0], ( *n* , %)	948 (91.2)	1,922 (91.2)
[2.5–3.5], ( *n* , %)	92 (8.8)	186 (8.8)

Abbreviations: INR, international normalized ratio; SD, standard deviation.

a Primary treatment indication.

b *p* -Value < 0.001.

In total, 1,134 VKA users completed the vaccination program. This group differed in treatment indication, age, gender, and the percentage of acenocoumarol users compared with the group receiving only one vaccination ( Supplementary Table S2 , available in the online version).

### Anticoagulation Control in all Patients after the First Vaccination

In the standard intensity group (INR 2.0–3.0) there was a decrease of 8.9% in INRs within range after first vaccination ( Table 2 ), due to a significant increase of both supratherapeutic INRs (INR > 3.0) as well as subtherapeutic INRs (< 2.0). There was both an increased risk of supratherapeutic INR levels (OR = 1.34 [95% confidence interval [CI] 1.08–1.67], *p* = 0.008) and subtherapeutic levels (OR = 1.40 [95% CI 1.08–1.83], *p* = 0.012) after first vaccination ( Table 3 ).

**Table 2:** anticoagulation levels before and after the first vaccination in every vaccine recipient ( *n* = 3148)

	Prior vaccination	After first vaccination
Standard intensity [2.0–3.0]		
INR below range	390 (13.6%) b	495 (17.2%) b
INR in range	2,170 (75.6%) b	1,914 (66.7%) b
INR above range	310 (10.8%) b	461 (16.1%) b
High intensity [2.5–3.5]		
INR below range	70 (25.2%)	66 (23.7%)
INR in range	185 (66.5%)	166 (59.7%)
INR above range	23 (8.3%) b	46 (16.5%) b
INR level (mean, SD)	2.50 (0.57) b	2.55 (0.70) b
Phenprocoumon tablets (mean, SD)	0.49 (0.22) b	0.49 (0.22) b
Acenocoumarol tablets (mean, SD)	1.78 (0.78)	1.77 (0.78)
INR ≥ 5	14 (0.4%)	20 (0.6%)
Significant dose adjustment a	31 (1.0%)	33 (1.0%)

Abbreviations: INR, international normalized ratio; SD, standard deviation.

a Significant dose adjustment is defined as a dose adjustment of 10% or more.

b *p* -Value < 0.05, calculated by McNemar's test or paired *t* -tests.

**Table 3:** Risk of INR out of range after vaccination: case-crossover analysis

	Before and after vaccination in every recipient ( *n* = 3,148)			Before and after first vaccination in subgroup ( *n* = 1,134) a			After first and second vaccination in subgroup ( *n* = 1,134) a			Before vaccination and after second vaccination in subgroup ( *n* = 1,134) a		
	OR	95% CI	*p* -Value	OR	95% CI	*p* -Value	OR	95% CI	*p* -Value	OR	95% CI	*p* -Value
Standard intensity												
INR in range	Reference			Reference			Reference			Reference		
Below range	**1.35**	1.18–1.56	**< 0.001**	**1.34**	1.08–1.67	**0.008**	0.93	0.76–1.14	0.50	1.23	0.987–1.535	0.065
Above range	**1.51**	1.30–1.76	**< 0.001**	**1.40**	1.08–1.83	**0.012**	0.90	0.70–1.15	0.32	1.27	0.971–1.649	0.082
High intensity												
INR in range	Reference			Reference			Reference			Reference		
Below range	0.90	0.51–1.54	0.78	0.90	0.51–1.54	0.78	0.80	0.44–1.44	0.46	0.77	0.45–1.32	0.34
Above range	**2.29**	1.22–4.28	**0.010**	**3.25**	1.06–9.97	**0.039**	1.02	0.52–2.22	0.85	**3.25**	1.06–9.97	**0.027**

Abbreviations: CI, confidence interval; INR, international normalized ratio; OR, odds ratio calculated using conditional logistic regression; VKA, vitamin K antagonist.

a Subgroup is defined as VKA users who completed the vaccination program.

In the high-intensity group (INR 2.5–3.5), VKA users were also more likely to have an INR above range after the first vaccination ( Table 2 ). The risk of a supratherapeutic INR was 3.5 times higher after first vaccination (OR 3.50 [95% CI 1.15–10.63], *p* = 0.027). A subtherapeutic INR after vaccination was as often observed as prior to vaccination.

Overall, the mean INR was significantly higher after the first vaccination than before vaccination (mean INR [SD] before vs. after, 2.50 [0.57] vs. 2.54 [0.68], *p* = 0.001) receiving at least one vaccination. The percentage of INRs ≥ 5 prior to vaccination was similar to the percentage after vaccination. The difference in mean phenprocoumon and acenocoumarol dose can be found in Table 2 .

#### Anticoagulation Control before and after First Vaccination in Subgroups

In the subgroup of VKA users ( *n* = 2,355) who had an INR within range prior to vaccination, 30.8% had an INR outside their therapeutic range afterwards. In the standard intensity group ( *n* = 2,170), 329 (15.2%) had a subtherapeutic INR (INR < 2.0) and 330 (15.2%) had a supratherapeutic INR (INR > 3.0). In the high-intensity group ( *n* = 185), 36 (19.5%) had a subtherapeutic INR (INR < 2.5) and 30 (16.2%) had a supratherapeutic INR (INR > 3.5) after vaccination. The mean INR in VKA users who had an INR within range prior to vaccination was also higher after the first vaccination (mean INR [SD] 2.48 [0.50] vs. 2.55 [0.66], *p* < 0.001). VKA users with a poor TTR ( *n* = 1,041) were more likely to have an INR out of range compared with patients with a TTR > 60% after the first vaccination (653 (31.3%) vs. 408 (39.2%), *p* = 0.001). In patients who had their INR measured within 14 days ( *n* = 2,706), similar results as the main analysis were seen.

### Anticoagulation Control in Patients Who Completed the Vaccination Program

The results after the first and second vaccination of the patients who completed the vaccination ( *n* = 1,334) are shown in Tables 3 and 4 . The percentages of INRs within range after first vaccination and second vaccination were similar. Likewise, no increased risk was observed for reaching an INR below or above range in both groups ( Table 4 ). The mean INR after the second vaccination was similar to the first vaccination. However, an increase of significant dose adjustments was seen after the second vaccination (13 [1.1%] vs. 68 [6.0%], *p* < 0.001). Similar results were seen in VKA users who had an INR in range prior vaccination and in VKA users with an INR measurement of 14 days or shorter after vaccination.

**Table 4:** Anticoagulation levels before and after vaccination in patients who completed the vaccination program ( *n* = 1,134)

	Prior vaccination	After first vaccination	After second vaccination
Standard intensity [2.0–3.0], *n* = 1,031			
Below range	161 (15.6%) b c	212 (20.6%) c	195 (18.9%) b
INR in range	753 (73.0%) b c	667 (64.7%) c	694 (67.3%) b
Above range	117 (11.3%) b	152 (14.7%) b	142 (13.8%)
High intensity [2.5–3.5], *n* = 103			
Below range	35 (34.0%)	28 (27.2%)	27 (26.2%)
INR in range	60 (58.3%)	54 (52.4%)	58 (56.3%)
Above range	8 (7.8%) b	21 (20.4%) b	18 (17.5%)
INR level (mean, SD)	2.47 (0.58)	2.52 (0.71)	2.51 (0.69)
Phenprocoumon tablets (mean, SD)	0.48 (0.24) b	0.48 (0.24)	0.48 (0.24) b
Acenocoumarol tablets (mean, SD)	1.77 (0.84)	1.77 (0.84)	1.76 (0.84)
INR ≥ 5	6 (0.5%)	6 (0.5%)	8 (0.7%)
Significant dose adjustment a	11 (1.0%)	13 (1.1%)	68 (6.0%)

Abbreviations: INR, international normalized ratio; SD, standard deviation.

a Significant dose adjustment is defined as a dose adjustment of 10% or more.

b *p* -Value < 0.05.

c *p* -Value < 0.001 calculated by McNemar's test or paired *t* -tests.

#### Anticoagulation Control Prior Vaccination Compared with after Second Vaccination

In the standard intensity group, the percentage of an INR within target range was significantly lower after the second vaccination compared with prior vaccination (753 [73.0%] vs. 694 [67.3%], *p* = 0.004). The percentage of subtherapeutic INR (INR < 2.0) was significantly higher after second vaccination than prior vaccination (161 [15.6%] vs. 195 [18.9%], *p* = 0.041). In this group, the OR did not differ before and after the vaccination program ( Table 3 ).

In the high-intensity group, no difference was seen in the percentage of INRs within range after the vaccination program ( Table 4 ). In the high-intensity group, the risk of supratherapeutic INR levels was 3.25 times higher after completing vaccination compared with prior vaccination (OR 3.25 [95% CI 1.06–9.97], *p* = 0.027) ( Table 3 ). Comparable results were seen in VKA users who had an INR in range prior vaccination and in VKA users with an INR measurement of 14 days or shorter after vaccination.

### Sensitivity Analyses

Sensitivity analyses showed that the percentage of INRs out of range was higher after the first vaccination, irrespective of the chosen baseline INR ( Supplementary Tables S3 and S4 , available in the online version). Patients with the most stable INR and standard intensity ( *n* = 603) experienced a decrease from 83.9 to 73.0% of INRs in range after the first vaccination ( *p* < 0.001). The risk of subtherapeutic (OR 1.35 [95% CI 1.17–1.55], *p* < 0.001) and supratherapeutic INR levels (OR 1.54 [95% CI 1.32–1.79], *p* < 0.001) were both increased after the first vaccination. In the high-intensity group ( *n* = 27), we did not observe a difference in the percentage of INRs in range after vaccination. However, the risk of supratherapeutic INR levels was increased after the first vaccination (OR 2.29 [95% CI 1.22–4.28], *p* = 0.01).

The mean INR level was significantly higher after the first vaccination, irrespective of intensity. In VKA users who completed the vaccination program, the high-intensity group had a significantly higher INR level after the first and second vaccination (2.68 [0.64] vs. 2.99 [0.95], *p* = 0.007 and 2.68 [0.64] vs. 2.92 [0.74], *p* = 0.01). After we stratified by VKA type, the results followed the main analysis.

## Discussion

Our research aimed to study whether BNT162b2 affects anticoagulation control in outpatients using VKA. Our results indicate that COVID-19 vaccination with BNT162b2 has a significant negative effect on anticoagulation control since 33.3% of patients had an INR out of range after the first vaccination compared with 24.4% prior to vaccination. This negative effect was also observed in the most stable VKA users and VKA users who were within range prior to the first vaccination. Nevertheless, BNT162b2 did not result in an increase of the percentage of INR ≥ 5.

There are several explanations for the effect of vaccination on anticoagulation control. Systemic reactions, including fever and chills, occur in up to 50% of vaccine recipients receiving the BNT162b2 vaccine. 15 However, these systemic reactions, are more frequently reported after the second BNT162b2 vaccination. 15 24 In contrast, the effect of BNT162b2 on anticoagulation control was less pronounced after the second vaccination. This finding, in combination with the increased percentage of dose adjustments, could indicate doctors' anticipation on the effects of vaccination making them dose differently for the second vaccination. Finally, patients themselves could have decided to decrease the dosage in the days following COVID-19 vaccination as they might be afraid for bleeding complications after intramuscular injection. This could result in a higher percentage of subtherapeutic INRs after vaccination.

Another explanation might be the inhibition of cytochrome p-450 caused by vaccination, which is seen in laboratory studies in mice receiving the DTP vaccine. 25 26 This group of enzymes is responsible for the metabolizing of acenocoumarol and, to a lesser extent, phenprocoumon. 27 A third explanation could be that the modified ribonucleic acid (RNA) encoding the SARS-CoV-2 full-length spike directly affects coagulation. The presence of spike protein S1 can result in structural changes in prohemostatic proteins. 28 In addition, messenger RNA (mRNA) vaccines can influence coagulation due to excessive extracellular RNA interacting with coagulation factors. 29

The possible effects of vaccines on anticoagulation control remain debated. Several prospective studies have examined the effect of the influenza vaccine on anticoagulation control. However, their results were conflicting, they had small sample sizes, and none of them were population-based. 30 Therefore, they might not be generalizable to all adults using VKA. A large retrospective study looked into different vaccines and did not detect any difference of clinical importance in mean INR after vaccination nor any tendency for INR measurements to be outside the therapeutic range. 31 However, their source data made it impossible to know the therapeutic indication and target range of their study population. The current study provides new insights into this debate by observing the immediate effects of BNT162b2 on INR stability in outpatient VKA users.

Our study has several strengths. First, by using the electronic patient files from four large anticoagulation clinics, we were able to acquire INR results of over 3,000 patients before and after vaccination. This large sample size made it possible to examine subgroups and perform several sensitivity analyses. Second, our study is population-based and thereby giving our results more generalizability than the previously mentioned studies. Collecting INR results both from VKA users who were fully vaccinated and from VKA users who only received the first vaccination minimized selection bias. Interestingly, patients who were fully vaccinated were younger and more often female than those who had received only one vaccination. This is an unusual observation as the Dutch COVID-19 vaccine program invites people first starting with the eldest. One possible explanation is that older people dropped out of the vaccination program more often, which might have been the case if they experienced side effects or complications. Thanks to our study design, we were able to study this phenomenon. Our final strength is that we included acenocoumarol as well as phenprocoumon users. In both users, an immediate effect of BNT162b2 was seen. Compared with the international, frequently used warfarin (half-life 40 hours), acenocoumarol has a relatively short half-life (11 hours) and phenprocoumon a relatively long half-life (140 hours). 32 As the results were alike in acenocoumarol and phenprocoumon users, the negative immediate effect is probably similar for warfarin users.

Our study also has limitations. The first limitation is that we only included patients who received a BNT162b2 vaccine. Still BNT162b2 is the most frequently used vaccine in Europe, with 69% of the Dutch vaccine recipients vaccinated with BNT162b2. However, caution is needed to generalize our results to the other vaccines, as not every COVID-19 vaccination is an mRNA vaccine. 17 33 Further research should look into the effects of other vaccine types. Our second limitation is the use of a surrogate variable for bleeding complications, namely an INR ≥ 5. We could not make any firm conclusions on registered complications, due to the heterogeneity between anticoagulation clinics in registering complications and the low number of complications on the different time points (data not shown). Therefore, we choose an INR ≥ 5 as surrogate marker. The percentage of INR ≥ 5 prior to the first vaccination was similar to the percentage after vaccination, suggesting that the risk of bleeding after vaccination is low. Our third limitation is that we cannot exclude the possibility that the negative effect on anticoagulation control was due to dose adjustments to avoid complications. However, vaccination is deemed safe when the INR < 3.5, so dose adjustment should not have been necessary for vaccination alone. Finally, our study population is older than the average Dutch VKA user, whose mean age is 73 years. 34 It is unknown whether elderly patients are more prone to be affected by external factors such as vaccines, even though elderly patients usually have a higher TTR than younger patients. 23 Future studies should also include younger patients to investigate whether the negative effect of COVID-19 vaccination persists in this patient population.

The findings of our study have implications for the management of VKA patients enrolling in a vaccination program. Dutch anticoagulation clinics have been intensely monitoring patients to identify those with INR values over 3.5. In these patients, necessary dose adjustment following INR results took place before vaccination. In patients with an INR within range before vaccination, 30% were outside range after the first vaccination. Therefore, an INR in range before vaccination is no predictor of INR stability during the vaccination program. Nevertheless, most vaccine recipients stayed in range during the COVID-19 program. Still, even a relatively small effect on anticoagulation control can be meaningful on a population level. Therefore, it is our opinion that frequent INR monitoring shortly after vaccination is advisable.

Although direct oral anticoagulant (DOAC) is the first-line treatment for atrial fibrillation and venous thrombosis, many patients are still treated with VKAs as second-line treatment or because they did not switch to a DOAC. One can postulate that switching VKA to DOAC before COVID-19 vaccination is beneficial for patients without contraindications for DOACs. We think that DOAC users do not experience clinically relevant direct effects of COVID-19 vaccination on the level of anticoagulation, although this has not been studied.

To conclude, our results indicate an immediate negative effect of BNT162b2 on anticoagulation control in patients treated with VKAs. Therefore, it is advisable to monitor the INR shortly after vaccination, even in stable patients.

## Notes

**What is known about this topic?**

- Any systemic event such as illness, fever, or physical stress can decrease anticoagulation control in VKA users.
- Almost 50% of recipients of COVID-19 vaccine experience systemic effects of the vaccination.
- In patients using vitamin K antagonists (VKA), the effects of COVID-19 vaccine on the anticoagulation control is uncertain.

**What does this paper add?**

- BNT162b2 is associated with an immediate detrimental effect on anticoagulation control in VKA users.
- One-third of VKA users had an INR out of range after the first vaccination compared with 24.4% prior vaccination.
- This study provides insight into the effects of BNT162b2 on anticoagulation control by using real-world data of more than 3,000 VKA users.
