# Supplementary material for: The Immediate Effect of COVID-19 Vaccination on Anticoagulation Control in Patients Using Vitamin K Antagonists
Source: Thromb Haemost. 2022 Mar 4;122(3):377–85. doi: 10.1055/s-0042-1742628 (PMC8899332; doi:10.1055/s-0042-1742628)
Supplement: Supplementary file 1 — Supplementary Material [file 10-1055-s-0042-1742628-s210447.pdf]

Supplementary Appendix A

Consortium Members of the Dutch COVID & Thrombosis Coalition

Amsterdam University Medical Center

- Location AMC:
  Prof. Dr. D. van de Beek, neurologist
  Dr. M. C. Brouwer, neurologist
  Drs S. de Bruin, phd-candidate
  Dr. M. Coppens, internist vascular medicine
  Drs. N. van Es, PhD candidate
  Drs. T. F. van Haaps, PhD-candidate Department of vascular medicine
  Prof. dr. N. P. Juffermans, intensivist
  Dr. M. C. A. Muller, intensivist
  Prof. Dr. A. P. J. Vlaar, intensivist
- Location VUMC:
  Prof. dr. C. M. P. M. Hertogh, nursing home specialist
  Prof. Dr. L. M. A. Heunks, professor intensive care
  Drs. J. G. Hugtenburg, pharmacologist
  Dr. J. van Kooten, nursing home specialist
  Dr. E. J. Nossent, pulmonologist
  Prof. Dr. Y. Smulders, internist
  Dr. P. R. Tuinman, intensivist
  Dr. A. Vonk Noordegraaf, pulmonologist

Amphia Hospital

Dr. M. J. J. H Grootenboers, pulmonologist
Dr. C van Guldener, internist
Dr. M. Kant, pulmonologist

Argos Zorggroep

Drs. A. Lansbergen, physiotherapist

Erasmus Medical Center

Drs. J. van den Akker, intensivist
Dr. R. Bierings, cellular biologist, Department Haematology
Dr. H. Endeman, intensivist
Dr. M. Goeijenbier, internal medicine, Department Viroscience
Dr. N. G. M. Hunfeld, hospital pharmacist
Prof. dr. E. C. M. van Gorp, infectious diseases specialist, Department Viroscience
Prof. dr. D. A. M. P. J. Gommers, intensivist
Prof. dr. M.P.G. Koopmans, internist, Department Viroscience
Dr. M. J. H. A. Kruip, haematologist
Prof. dr. T. Kuiken, professor of comparative pathology, Department Viroscience
Drs. T. Langerak, PhD-candidate, Department Viroscience
Prof. Dr. Leebeek, professor of hemostasis and thrombosis
Dr. M. N. Lauw, haematologist, Department Haematology
Prof. dr. M. P. M. de Maat, head of Biochemistry of Haemostasis and Thrombosis
Drs. D. Noack, PhD-candidate i.o. Department Viroscience
Drs. M.S. Paats, pulmonologist
Drs. M.P. Raadsen, PhD-candidate Department Viroscience
Dr. B. Rockx, assistant professors, Department Viroscience
Dr. C. Rokx, infectious diseases specialist
Dr. C. A. M. Schurink, infectious diseases specialist

 © 2022. The Author(s).

Drs. K. Tong-Minh, PhD-candidate Department Viroscience
Dr. L. van den Toorn, pulmonologist
Dr. C. A. den Uil, Cardiologist-Intensivist
Drs. C. Visser, PhD-candidate, Department haematology

Farmadam

Drs. F. Boutkourt, PhD-candidate
Drs. T. Roest, pharmacist

Flevoziekenhuis

Dr. R. A. Douma, infectious diseases specialist
Drs. L. R. de Haan, PhD-candidate
Dr. M. ten Wolde, internist vascular medicine

Hospital de Gelderse Vallei

Dr. R. H. H. Bemelmans, internist
Dr. B. Festen, intensivist

Ikazia Hospital

Dr. S. Stads, intensivist

Jeroen Bosch Hospital

Dr. C. P. C. de Jager, intensivist
Dr. K.S. Simons, intensivist

Leiden University Medical Center

Drs. M.L. Antoni, cardiologist, Department of cardiology
Dr. M. H. Bos, biochemicus, associate professor, Department of Medicine - Thrombosis and Hemostasis
Drs. J. L. I. Burggraaf, PhD-candidate, Department of Clinical Epidemiology
Prof. S. C. Cannegieter, clinical epidemiologist, Department of Medicine - Thrombosis and Hemostasis and Department of Department of Clinical Epidemiology
Prof. dr. H. C. J. Eikenboom, haematologist/internist vascular medicine, Department of Medicine - Thrombosis and Hemostasis
Dr. P. L. den Exter, vascular medicine specialist, Department of Medicine - Thrombosis and Hemostasis
Dr. J. J. M. Geelhoed, pulmonologist, Department of Pulmonology
Prof. dr. M. V. Huisman, internist vascular medicine, Department of Medicine - Thrombosis and Hemostasis
Prof. E. de Jonge, internist-intensivist, Department of Intensive Care Medicine
Drs. F. H. J. Kaptein, PhD-candidate, Department of Medicine - Thrombosis and Hemostasis
Dr. F. A. Klok, internist vascular medicine, Department of Medicine - Thrombosis and Hemostasis
Dr. L. J. M. Kroft, radioloog, Department of Radiology
Dr. W. M. Lijfering, Clinical epidemiologist, department of medicine – Thrombosis and Hemostasis
Drs. L. Nab, PhD-candidate, Department of Clinical Epidemiology
Dr. M. K. Ninaber, pulmonologist, Department of Pulmonology
Prof. dr. H. Putter, statistician, Department of Biomedical Data Sciences
Drs. S. R. S. Ramai, pulmonologist, Department of Pulmonology
Dr. A. M. da Rocha Rondon, Postdoctoral researcher, Department of Medicine - Thrombosis and Hemostasis
Dr. A. H. E. Roukens, infectious diseases specialist, Department of infectious diseases
Drs. M. A. M. Stals, PhD-candidate, Department of Medicine - Thrombosis and Hemostasis
Prof. dr. H .H. Versteeg, cellular biologist, Department of Medicine - Thrombosis and Hemostasis
Dr. H. W. Vliegen, cardiologist, Department of cardiology
Dr. B. J. M. van Vlijmen, cellular biologist, associate professor, Department of Medicine - Thrombosis and Hemostasis

Maastricht University Medical Center

Drs. T. van de Berg, PhD-candidate
Drs. R. Bruggemann, PhD-candidate
Dr. B. C. T. van Bussel, internist-intensivist
Prof. dr. H. ten Cate, Internist
Dr. A. Ten Cate-Hoek, Clincial epidemiologist and medical director of thrombosis service Maastricht

 © 2022. The Author(s).

Prof. dr. T. M. Hackeng, biochemist
Dr. ir. Y. Henskens, clinical chemist
Drs. A. Hulshof, PhD-candidate
Drs. M. Mulder, PhD-candidate
Drs. R. H. Olie, internist vascular medicine
Prof. dr. L. Schurgers, biochemist
Dr. B. Spaetgens, internist subspecialized in geriatrics
Dr. H. Spronk, biochemist
Prof. dr. M. A. Spruit, executive board member Ciro and Professor in Rehabilitation
Dr. K. Winckers, internist vascular medicine

Maxima Medical Center

Dr. L. Nieuwenhuizen, haematologist

Medical Center Leeuwaarden

Drs. B. Franken, haematologist
Dr. I. M. Schrover, internist vascular medicine
Drs. E. G. M. de Waal, haematologist

Medical Center Twente

Dr. A. Beishuizen, intensivist
Dr. A. Cornet, intensivist
Dr. J. Krabbe, clinical biochemist

Radboud University Medical Center

Prof. dr. K. Kramers, professor medical safety
Dr. J. Leentjens, internist vascular medicine
Dr. Q. de Mast, infectious diseases specialist
Prof. dr. S. Middeldorp, internist vascular medicine

Reinier de Graaf Gasthuis Hospital

Dr. R. E. Brouwer, haematologist
Dr. J. L. J. Ellerbroek, infectious diseases specialist
Drs. J. Tijmensen, haematologist

Rijnstate Hospital

Dr. M. M. C. Hovens, internist vascular medicine
Dr. E. A. N. Oostdijk, intensivist
Drs. B. D. Westerhof, anaesthesiologist-intensivist

Rode Kruis Hospital

Dr. L. M. Faber, Haematologist

Sanquin Research, Amsterdam

Dr. M. van den Biggelaar, head of Laboratory of Proteomics, Department of Molecular and Cellular Hemostasis
Prof. Dr. J. C. M. Meijers, biochemist (and Amsterdam University Medical Centers)
Prof. dr. J. Voorberg, molecular en cellular biologist (and Amsterdam University Medical Centers)

St Fransiscus Gasthuis & Vlietland Hospital

Dr. M. E. Kevenaar, internist
Drs. Y. L. Soei, internist
Dr. E. J. Wils, intensivist

St. Jansdal Hospital

Dr. F. N. Croles, haematologist

Synapse Research Institute

Dr. B. de Laat, biochemist, director

 © 2022. The Author(s).

Tergooi Hospital

Prof. Dr. P. W. Kamphuisen, internist vascular medicine
Dr. R. Vink, intensivist

University Medical Center Groningen

Prof. dr. T. Lisman, biochemist
Prof. dr. K. Meijer, haematologist, Department Haematology
Dr. Y. I. G. van Tichelaar, internist

University Medical Center Utrecht

Prof dr. O. L. Cremer, anesthesiologist-intensivist
Dr. G. Geersing, general practicioner, Julius Center, Department primary care
Prof dr. H. A. H. Kaasjager, internist vascular medicine
Dr. N. Kusadasi, haematologist-intensivist
Dr. A. Huisman, clinical biochemist
Dr. C. Maas, principal investigator coagulation & fibrinolysis
Dr. M. Nijkeuter, internist vascular medicine
Prof. dr. R.E.G. Schutgens, haematologist, Van Creveldkliniek
Dr. R. T. Urbanus, biochemist, Van Creveldkliniek
Dr. J. Westerink, internist vascular medicine

Wilhelmina Hospital Assen

Dr. H. J. Faber, internist-intensivist

Zaans Medical Center

Drs. S. C. E. Koster, anesthesiologist-intensivist

Zuyderland Hospital

Dr. P. van Montfort, resident internal medicine
Dr. D. J. L. van Twist, internist vascular medicine

Supplementary Table S1 Clinical characteristics patients who completed the vaccination program ($n = 1{,}134$)

	Phenprocoumon	Acenocoumarol
Patients (*n*, %)	333 (29.4)	801 (70.6)
Age (mean, SD)	84.6 (12.2)[b]	86.1 (11.2)[b]
Men (*n*, %)	114 (34.2)	301 (37.6)
Treatment indication[a]		
Atrial fibrillation (*n*, %)	253 (76.0)	632 (78.9)
Venous thrombosis (*n*, %)	43 (12.9)	80 (10.0)
Mechanical heart valves (*n*, %)	11 (3.3)	35 (4.4)
Vascular surgery (*n*, %)	8 (2.4)	9 (1.1)
Ischemic heart disease (*n*, %)	1 (0.3)	6 (0.7)
Other (*n*, %)	17 (5.1)	39 (4.9)
Target INR		
[2.0–3.0], (*n*, %)	303 (91.0)	728 (90.9)
[2.5–3.5], (*n*, %)	30 (9.0)	73 (9.1)

Abbreviations: INR, international normalized ratio; SD, standard deviation.
[a]Primary treatment indication.
[b]p-Value <0.05.

 © 2022. The Author(s).

Supplementary Table S2 Clinical characteristics of patients who completed the vaccination program vs patients receiving only one vaccination

	Patients with only one vaccination	Patients who completed the vaccination program
Patients (*n*, %)	2,014 (64.0)	1,134 (36.0)
Age (mean, SD)	87.3 (6.5)[c]	85.7 (11.5)[c]
Men (*n*, %)	965 (47.9)[c]	415 (36.6)[c]
Acenocoumarol (*n*, %)	1,307 (64.9)[c]	801 (70.6)[c]
Treatment indication[a]		
Atrial fibrillation (*n*, %)	1,643 (81.6)[b]	885 (78.0)[b]
Venous thrombosis (*n*, %)	152 (7.5)[b]	123 (10.8)[b]
Mechanical heart valves (*n*, %)	91 (4.5)	46 (4.1)
Vascular surgery (*n*, %)	37 (1.8)	17 (1.5)
Ischemic heart disease (*n*, %)	11 (0.5)	7 (0.6)
Other (*n*, %)	80 (4.0)	56 (4.9)
Target INR		
Low intensity [2.0–3.0], (*n*, %)	1,839 (91.3)	1,031 (90.9)
High intensity [2.5–3.5], (*n*, %)	175 (8.7)	103 (9.1)

Abbreviations: INR, international normalized ratio; SD, standard deviation.
[a]Primary treatment indication.
[b]p-Value < 0.05.
[c]p-Value < 0.001.

Supplementary Table S3 Sensitivity analyses second INR and 1 month before vaccination and average INR as baseline in every vaccine recipient

**Second INR before vaccination as baseline**		
	**Prior vaccination**	**After first vaccination**
Standard intensity [2.0–3.0]		
INR below range (*n*, %)	327 (11.4)[a]	495 (17.2)[a]
INR in range (*n*, %)	2,135 (74.7)[a]	1,914 (66.7)[a]
INR above range (*n*, %)	396 (13.9)[a]	461 (16.1)[a]
High intensity [2.5–3.5]		
INR below range (*n*, %)	40 (14.5)[a]	66 (23.7)[a]
INR in range (*n*, %)	182 (65.9)	166 (59.7)
INR above range (*n*, %)	54 (19.6)	46 (16.5)
**INR 1 month before vaccination as baseline**		
	**Prior vaccination**	**After first vaccination**
Standard intensity [2.0–3.0]		
INR below range (*n*, %)	322 (11.3)[a]	495 (17.2)[a]
INR in range (*n*, %)	2,095 (73.4)[a]	1,914 (66.7)[a]
INR above range (*n*, %)	437 (15.3)	461 (16.1)
High intensity [2.5–3.5]		
INR below range (*n*, %)	48 (17.4)[a]	66 (23.7)[a]
INR in range (*n*, %)	178 (64.5)	166 (59.7)
INR above range (*n*, %)	50 (18.1)	46 (16.5)

Abbreviation: INR, international normalized ratio.
[a]p-Value < 0.05, calculated by McNemar's test.

 © 2022. The Author(s).

Supplementary Table S4 Sensitivity analyses second INR and 1 month before vaccination and average INR as baseline in vaccine recipients who completed the vaccine program

Second INR before vaccination as baseline			
	**Prior vaccination**	**After first vaccination**	**After second vaccination**
Standard intensity [2.0–3.0]			
INR below range (*n*, %)	138 (13.4)[a,b]	212 (20.6)[a]	195 (18.9)[b]
INR in range (*n*, %)	758 (73.8)[a,b]	667 (64.7)[a]	694 (67.3)[b]
INR above range (*n*, %)	131 (12.8)	152 (14.7)	142 (13.8)
High intensity [2.5–3.5]			
INR below range (*n*, %)	19 (18.4)	28 (27.2)	27 (26.2)
INR in range (*n*, %)	64 (62.1)	54 (52.4)	58 (56.3)
INR above range (*n*, %)	20 (19.4)	21 (20.4)	18 (17.5)
**INR 1 month before vaccination as baseline**			
	**Prior vaccination**	**After first vaccination**	**After second vaccination**
Standard intensity [2.0–3.0]			
INR below range (*n*, %)	134 (13.1)[a,b]	212 (20.6)[a]	195 (18.9)[b]
INR in range (*n*, %)	734 (71.5)[a,b]	667 (64.7)[a]	694 (67.3)[b]
INR above range (*n*, %)	158 (15.4)	152 (14.7)	142 (13.8)
High intensity [2.5–3.5]			
INR below range (*n*, %)	15 (14.6)[a,b]	28 (27.2)[a]	27 (26.2)[b]
INR in range (*n*, %)	77 (74.8)[a,b]	54 (52.4)[a]	58 (56.3)[b]
INR above range (*n*, %)	11 (10.7)	21 (20.4)	18 (17.5)

Abbreviation: INR, international normalized ratio.
[a] p-Value < 0.05, calculated by McNemar's test or paired t-tests.

 © 2022. The Author(s).
